# Comparative performance of CMV IgM and IgG avidity assays for diagnosing a recent primary infection in the first trimester of pregnancy

**DOI:** 10.1128/jcm.00545-26

**Published:** 2026-08-10

**Authors:** Nicolas Veyrenche, Jacques Fourgeaud, Meryem Mekouar, Tiffany Guilleminot, Nicolas Bourgon, Yves Ville, Marianne Leruez-Ville

**Affiliations:** 1 Virology Unit – National reference laboratory for herpes viruses - Hôpital Necker Enfants Malades, AP-HP, Hôpital Necker Enfants Malades 246596, Paris, France; 2 Université Paris Cité, FETUS, Paris, France; 3 Obstetrics, Fetal Medicine Surgery and Imaging Unit - Hôpital Necker Enfants Malades, GHU Paris Centre, AP-HP 26952 , Paris, France; St Jude Children's Research Hospital, Memphis, Tennessee, USA

**Keywords:** congenital cytomegalovirus, CMV, serology, IgM and IgG avidity, CMV screening, maternal primary infection

## Abstract

First-trimester (T1) cytomegalovirus (CMV) screening relies on IgG, IgM, and IgG avidity testing, but assay performance may vary according to the infection timing. To evaluate the ability of two IgM and three IgG avidity assays to detect maternal primary CMV infection (MPI) occurring in T1 or the periconceptional period. We analyzed 176 serum samples from 94 pregnant women with precisely dated MPI based on seroconversion. Samples were tested using two IgM assays (LIAISON CMV IgM II, Alinity i CMV IgM) and three IgG avidity assays (VIDAS CMV IgG Avidity II, LIAISON CMV IgG Avidity II, Alinity i CMV IgG avidity). For detection of MPI within 4 months, the IgM sensitivity was 99% (122/123) with LIAISON and 92% (94/102) with Alinity i. IgG avidity sensitivity for excluding MPI <4 months was 99% (124/125) with LIAISON, 98% (121/123) with VIDAS, and 92% (111/120) with Alinity i. The positive predictive value of low avidity was highest with VIDAS, followed by LIAISON and Alinity i. Retesting intermediate-avidity results with a second assay improved the estimation of MPI timing. All three avidity assays reliably excluded T1 MPI. Alinity i showed reduced sensitivity for ruling out periconceptional infection, supporting confirmatory testing in equivocal cases.

**IMPORTANCE:**

In the context of universal first-trimester cytomegalovirus (CMV) screening and the demonstrated efficacy of valacyclovir to prevent vertical transmission, reliable identification of recent maternal primary infection has become crucial. In this study, we compared the performance of two CMV IgM kits (LIAISON CMV IgM II [DiaSorin]; Alinity i CMV IgM [Abbott Diagnostics]) and three CMV IgG avidity kits (LIAISON CMV IgG Avidity II [DiaSorin]; VIDAS CMV IgG Avidity II [Biomérieux]; Alinity i CMV IgG avidity [Abbott Diagnostics]). Our study assesses the performance of commonly used CMV IgM and IgG avidity assays in a large cohort of pregnant women with precisely dated primary infections, a setting rarely available for assay validation. We provide gestational age-specific performance data and highlight the limited positive predictive value of intermediate-IgG avidity results. Based on these findings, we propose a pragmatic diagnostic algorithm aimed at reducing misclassification and inappropriate clinical management.

## INTRODUCTION

Cytomegalovirus (CMV) is the leading cause of congenital infection, with a neonatal prevalence of 0.40%–0.60% in Western Europe and the United States (1). In Europe, more than half of congenital cytomegalovirus (cCMV) infections follow maternal primary infection, and in the USA, it was recently reported that primary infections represent 70% of congenital infections (2, 3). When primary infection occurs during the periconceptional period or the first trimester (T1), the risks of fetal transmission are estimated at 21%–22% and 37%–40%, respectively (4–6), and 30%–40% of infected fetuses subsequently develop long-term sensorineural sequelae (1, 4, 6).

Since 2020, the management of early maternal CMV primary infection has been altered by encouraging findings suggesting the efficacy of valacyclovir in reducing the risk of vertical transmission after maternal infection acquired both in the first trimester and in the periconceptional period (7–10). In some European countries, such as Italy and France, healthcare authorities recommend routine CMV serology screening during the first trimester of pregnancy (11, 12). In addition, certain obstetrics and gynecology professional societies, for example in Greece, Israel, and Canada, recommend this screening (13–15). Accurate identification of women eligible for antiviral prophylaxis is, therefore, critical, underscoring the need to validate robust laboratory tools and diagnostic algorithms.

Current screening strategies rely on CMV IgG and IgM testing. However, since IgM tests are known to lack specificity, it is mandatory to test for IgG avidity in sera with concomitant IgG and IgM positivity to help determine the timing of the primary infection (16, 17). The sensitivity of CMV screening during pregnancy relies on the sensitivity of IgM assays used. On the other hand, since around 1.5% to 3% of pregnant women in their first trimester have CMV-positive IgM (1, 18), the performance of IgG avidity assays to properly discriminate between recent (less than 4 months) and later-onset (more than 4 months) maternal infections is crucial. Knowing that only 10% of pregnant women with positive IgM have a recent primary infection (18), in this study, we compared the performance of two CMV IgM kits (LIAISON CMV IgM II [DiaSorin]; Alinity i CMV IgM [Abbott Diagnostics]) that account for more than 50% of the market in France (based on the participation of French laboratories in external quality assurance programs) and those of the three CMV IgG avidity kits mostly used in Europe and the US (LIAISON CMV IgG Avidity II [DiaSorin]; VIDAS CMV IgG Avidity II [Biomérieux]; Alinity i CMV IgG avidity [Abbott Diagnostics]). Using consecutive serum samples derived from pregnant women with a first-trimester primary infection of a precisely known timing, we aimed to assess assay sensitivity for early infection, estimate the positive predictive value of IgG avidity for recent primary infection, and define the optimal management of intermediate avidity results.

## MATERIALS AND METHODS

### CMV primary infection samples

This retrospective, single-center study was conducted at Necker–Enfants Malades Hospital in a population of pregnant women referred to the Prenatal Diagnosis Unit for a primary CMV infection occurring either during the periconceptional period or in the first trimester of pregnancy. All women underwent CMV serological testing in the hospital’s virology laboratory. Eligible cases were defined as maternal primary CMV infections with seroconversion that could be precisely dated. The timing of infection was determined according to serological criteria. In women whose first serum sample showed positive CMV IgM with negative IgG, followed by documented IgG seroconversion on a subsequent sample, the date of maternal primary infection was estimated as 10 days prior to the initial blood sampling. In cases of documented seroconversion between two serum samples, the date of infection was estimated as the midpoint between the last IgG-negative and the first IgG-positive sample, provided that the interval between the two samples was less than 4 weeks. All eligible cases recorded between 2012 and 2024 were retrieved from the laboratory information system.

A recent maternal primary CMV infection (MPI) was defined as an infection occurring within the previous 4 months. The periconceptional period was defined as the interval from 4 weeks before conception to 3 weeks of gestation (i.e., 1 week post-conception). The first trimester was defined as gestational weeks 4 to 14 (corresponding to 2 to 12 weeks post-conception).

### CMV IgG and IgM testing

CMV IgG and CMV IgM testing were performed on the Liaison XL platform with LIAISON CMV IgG II and LIAISON CMV IgM II (DiaSorin). IgG are positive when ≥ 14 U/mL. IgM are equivocal when ≥ 18 U/mL and <22 U/mL and positive when ≥ 22 U/mL. CMV IgG and CMV IgM testing were also performed with the Alinity i CMV IgG and Alinity i CMV IgM (Abbott Diagnostics). IgG are positive when ≥ 6 AU/mL. IgM are equivocal when ≥ 0.85 (index) and <1 (index) and positive when ≥ 1 (index).

### CMV IgG avidity testing

IgG avidity was measured with the VIDAS CMV IgG Avidity II (Biomérieux), the LIAISON CMV IgG Avidity II (Diasorin), and the Alinity i CMV IgG (Abbott) avidity assays. For the VIDAS assay, IgG avidity results were categorized as low, intermediate, or high based on the values of <0.40, ≥0.40 to < 0.65, and ≥0.65, respectively. For the LIAISON assay, IgG avidity results were categorized as low, intermediate, or high based on the values of <0.150, ≥0.150 to < 0.350, and ≥0.350, respectively. For the Alinity i assay, IgG avidity results were categorized as low, intermediate, or high based on the values of <50%, ≥50% to < 60%, and ≥60%, respectively.

IgG avidity values were interpreted according to the manufacturers’ recommendations, as previously summarized, except that the threshold value for high avidity with Liaison used in that study was not 0.250, as recommended by the manufacturer, but 0.350, as reported previously (5, 19). The VIDAS and LIAISON avidity results were obtained prospectively as part of the routine diagnosis, while results with the Alinity i avidity assay were obtained retrospectively by testing −20°C frozen samples.

Avidity assays were performed in sera with positive or equivocal IgM and IgG levels at least twice higher than the threshold value as recommended (20–22).

### Statistical analysis

The sensitivity, positive predictive value (PPV), and the 95% confidence interval (95% CI) were computed using GraphPad Prism 10.0.3 (GraphPad Prism Software Inc.). When comparing the sensitivity or PPV of diagnostic tests within unpaired samples, the Fisher’s exact test was used for pairwise comparisons. When comparing the sensitivity or PPV of diagnostic tests within paired samples, the McNemar test was used for pairwise comparisons. When multiple pairwise comparisons were performed, a Holm-Sidak correction was applied to Fisher’s exact test or to McNemar test. Statistical significance was defined as a *P*-value < 0.05. All statistical analyses were performed using GraphPad Prism 10.0.3 (GraphPad Prism Software Inc.).

## RESULTS

### Population

A total of 176 serum samples from 94 pregnant women with a precisely dated primary CMV infection (diagnosed in 35% by isolated IgM, followed by seroconversion, and in 65% by seroconversion between two samples collected less than 4 weeks apart) occurring during the first trimester or the periconceptional period were included. Forty-five women contributed a single sample, whereas 49 provided at least two consecutive samples. Samples were collected at a median of 62.5 days after infection onset (range 9–252 days; interquartile range [IQR], 34–136.5 days). Among those samples, 98.3% (173/176) were tested with the VIDAS CMV IgG Avidity II, 98.3% (173/176) with the LIAISON CMV IgG Avidity II, and 94.9% (167/176) with the Alinity i CMV IgG avidity assay.

As most women were referred to our center after the diagnosis of primary infection, initial IgM positivity had been detected in private laboratories using different assays: LIAISON in 37% of cases (35/94), Alinity i or Architect in 36% (34/94), Cobas (Roche) in 20% (19/94), and Beckman IgM in 6% (6/94).

### Sensitivity of IgM to diagnose a recent CMV primary infection

#### According to the interval between primary infection onset and sample collection

Cumulative IgM sensitivity was analyzed based on the time elapsed between maternal CMV primary infection (MPI) onset and serum sampling, with equivocal IgM results considered positive (Table 1). For the diagnosis of MPI within 3 months, IgM sensitivity was 94.7% (89/94) with the Alinity i assay and 100% (114/114) with the LIAISON assay. Within 4 months of MPI onset, the sensitivity was 92.2% (94/102) for Alinity i and 99.2% (122/123) for LIAISON.

**TABLE 1:** Sensitivity of IgM to diagnose a recent maternal primary CMV infection according to the time elapsed between sample collection and onset of primary infection

Time between sample collection and maternal CMV primary infection	Alinity i CMV IgM sensitivity % (*n/N*)^*a*^	LIAISON CMV IgM II sensitivity % (*n/N*)^*a*^	*P*-value
≤56 days (8 weeks)	100% (64/64) (95% CI: 94.3–100)	100% (78/78) (95% CI: 95.3–100)	*P >* 0.99
≤70 days (10 weeks)	96.1% (73/76) (95% CI: 89–98.9)	100% (94/94) (95% CI: 96.1–100)	*P =* 0.09
≤90 days (3 months)	94.7% (89/94) (95% CI: 88.2–97.7)	100% (114/114) (95% CI: 96.7–100)	*P =* 0.02
≤105 days (15 weeks)	92.9% (91/98) (95% CI: 86–96.5)	99.2% (117/118) (95% CI: 95.4–100)	*P =* 0.02
≤120 days (4 months)	92.2% (94/102) (95% CI: 85.3–96)	99.2% (122/123) (95% CI: 95.5–100)	*P =* 0.01
≤180 days (6 months)	88.8% (111/125) (95% CI: 82.1–93.2)	98% (145/148) (95% CI: 94.2–99.5)	*P =* 0.002

^*a*^ Sensitivity calculated as equivocal and positive CMV IgM/total percentage (*n/N*).

IgM sensitivity was significantly higher with the LIAISON assay than with the Alinity i assay when the interval between MPI onset and sampling was ≥3 months and ≤6 months (*P* = 0.02, 0.02, 0.01, and 0.002). No significant difference was observed when samples were collected within 3 months of MPI onset (Table 1).

When equivocal IgM results were considered negative, IgM sensitivity for diagnosing MPI within 3 and 4 months decreased to 92.6% (87/94) and 87.3% (89/102), respectively, for Alinity i and to 98.3% (112/114) and 95.1% (117/123), respectively, for LIAISON.

#### According to gestational age at sampling

Extrapolation of these data showed that IgM sensitivity for diagnosing a first-trimester MPI would have been 100% with the LIAISON assay regardless of gestational age at sampling (Table 2). With the Alinity i assay, the sensitivity would also have been 100% if samples had been collected at 7, 8, 10, or 12 weeks of gestation but decreased to 96.1% (73/76) when sampling occurred at 14 weeks. However, no significant difference was observed between the two assays (*P* > 0.05).

**TABLE 2:** Sensitivity of IgM to diagnose a recent maternal CMV primary infection (in the first trimester and/or periconceptional period) according to gestational age at sampling

	In the first trimester			In the first trimester and periconceptional period		
	Alinity i CMV IgM sensitivity % (*n/N*)^*a*^	LIAISON CMV IgM II sensitivity % (*n/N*)^*a*^	*P*-value	Alinity i CMV IgM sensitivity % (*n/N*)^*a*^	LIAISON CMV IgM II sensitivity % (*n/N*)^*a*^	*P*-value
Serology at 14 weeks	96.1% (73/76) (95% CI: 89-98.9)	100% (94/94) (95% CI: 96.1-100)	*P* = 0.09	92.9% (91/98) (95% CI: 86-96.5)	99.2% (117/118) (95% CI: 95.4-100)	*P* = 0.02
Serology at 12 weeks	100% (64/64) (95% CI: 94.3-100)	100% (78/78) (95% CI: 95.3-100)	*P* > 0.99	94.7% (89/94) (95% CI: 88.2-97.7)	100% (114/114) (95% CI: 96.7-100)	*P* = 0.02
Serology at 10 weeks	100% (47/47) (95% CI: 92.4-100)	100% (58/58) (95% CI: 93.8-100)	*P* > 0.99	96.4% (81/84) (95% CI: 90-99.0)	100% (103/103) (95% CI: 96.4-100)	*P* = 0.09
Serology at 8 weeks	100% (26/26) (95% CI: 87.1-100)	100% (32/32) (95% CI: 89.3-100)	*P* > 0.99	97.1% (67/69) (95% CI: 90-99.5)	100% (86/86) (95% CI: 95.7-100)	*P* = 0.20
Serology at 7 weeks	100% (19/19) (95% CI: 83.2-100)	100% (22/22) (95% CI: 85.1-100)	*P* > 0.99	100% (64/64) (95% CI: 94.3-100)	100% (78/78) (95% CI: 95.3-100)	*P* > 0.99

^*a*^ Sensitivity calculated as equivocal and positive CMV IgM/total percentage (*n/N*).

When considering the combined periconceptional and first-trimester period (Table 2), IgM sensitivity would have ranged from 99.2% to 100% with LIAISON and from 92.9% to 100% with Alinity i, assuming sampling at 7 and 14 weeks of gestation, respectively. IgM sensitivity was significantly higher with LIAISON when serology was performed at 12 or 14 weeks (*P* = 0.02), but not when testing was performed at 7, 8, or 10 weeks (*P* = 0.09, 0.20, and >0.99).

Taken together with the time-dependent cumulative sensitivity analysis (Table 1), these findings indicate that both assays perform similarly early after infection, whereas the LIAISON assay demonstrates greater robustness as the interval between infection and sampling increases.

### Sensitivity of avidity assays to detect a primary infection

#### According to the interval between infection onset and sampling

IgG avidity sensitivity for detecting a MPI within 3 months was 94.6% (106/112) with Alinity i and 100% with both LIAISON and VIDAS (Table 3). Within 4 months, the sensitivity was 92.5% (111/120) for Alinity i, 99.2% (124/125) for LIAISON, and 98.4% (121/123) for VIDAS.

**TABLE 3:** Sensitivity of IgG avidity to detect a maternal CMV primary infection according to the time elapsed between sample collection and onset of primary infection

Time between sample collection and maternal CMV primary infection	Alinity i CMV IgG avidity (A) sensitivity % (*n/N*)^*b*^	LIAISON CMV IgG Avidity II^*a*^ (B) sensitivity % (*n/N*)^*b*^	VIDAS CMV IgG Avidity II (C) sensitivity % (*n/N*)^*b*^	*P*-value
≤56 days (8 weeks)	98.7% (78/79) (95% CI: 93.2–99.9)	100% (80/80) (95% CI: 95.4–100)	100% (78/78) (95% CI: 95.3–100)	*P*_A vs B_ > 0.99 *P*_A vs C_ > 0.99 *P*_B vs C_ > 0.99
≤70 days (10 weeks)	97.9% (91/93) (95% CI: 92.6–99.6)	100% (96/96) (95% CI: 96.2–100)	100% (94/94) (95% CI: 96.1–100)	*P*_A vs B_ > 0.99 *P*_A vs C_ > 0.99 *P*_B vs C_ > 0.99
≤90 days (3 months)	94.6% (106/112) (95% CI: 88.8–97.5)	100% (116/116) (95% CI: 96.8–100)	100% (114/114) (95% CI: 96.7–100)	*P*_A vs B_ = 0.04 *P*_A vs C_ = 0.04 *P*_B vs C_ > 0.99
≤105 days (15 weeks)	94% (109/116) (95% CI: 88.1–97.1)	100% (120/120) (95% CI: 96.9–100)	99.2% (117/118) (95% CI: 95.4–100)	*P*_A vs B_ = 0.02 *P*_A vs C_ = 0.07 *P*_B vs C_ = 0.50
≤120 days (4 months)	92.5% (111/120) (95% CI: 86.4–96)	99.2% (124/125) (95% CI: 95.6–100)	98.4% (121/123) (95% CI: 94.3–99.7)	*P*_A vs B_ = 0.03 *P*_A vs C_ = 0.06 *P*_B vs C_ = 0.62

^*a*^ High-avidity threshold (LIAISON), ≥0.350.

^*b*^ Sensitivity calculated as low and intermediate avidity/total percentage (*n/N*).

No significant difference was observed among assays when the interval between infection and sampling was ≤70 days. When this interval was extended to ≤90 days, LIAISON and VIDAS demonstrated significantly higher sensitivity than Alinity i (*P* = 0.04). At ≤120 days, LIAISON remained significantly more sensitive than Alinity i (*P* = 0.03) (Table 3).

Using the high-avidity threshold (≥0.250) recommended by the manufacturer for LIAISON would have yielded sensitivities of 97.4% (113/116) and 95.2% (119/125) for MPI within 3 and 4 months, respectively, but without a statistically significant difference compared with the standard threshold (*P* = 0.25 and 0.12).

#### According to gestational age at sampling

Extrapolated analyses showed that IgG avidity sensitivity for diagnosing a first-trimester MPI would have been 100% (Table 4) with LIAISON and VIDAS, regardless of gestational age at sampling. With Alinity i, the sensitivity would have reached 100% at 7, 8, and 10 weeks but decreased to 98.7% (78/79) and 97.9% (91/93) at 12 and 14 weeks, respectively, without a significant difference between assays (*P* > 0.56) (Table 4).

**TABLE 4:** Sensitivity of IgG avidity to detect a recent maternal CMV primary infection (in the first trimester and/or periconceptional period) according to gestational age at sampling

	In the first trimester				In the first trimester and periconceptional period			
	Alinity i CMV IgG avidity (A) sensitivity % (*n/N*)^*b*^	LIAISON CMV IgG Avidity II^*a*^ (B) sensitivity % (*n/N*)^*b*^	VIDAS CMV IgG Avidity II (C) sensitivity % (*n/N*)^*b*^	*P*-value	Alinity i CMV IgG avidity (A) sensitivity % (*n/N*)^*b*^	LIAISON CMV IgG Avidity II^*a*^ (B) sensitivity % (*n/N*)^*b*^	VIDAS CMV IgG Avidity II (C) sensitivity % (*n/N*)^*b*^	*P*-value
Serology at 14 weeks	97.9% (91/93) (95% CI: 92.5–99.6)	100% (96/96) (95% CI: 96.2–100)	100% (94/94) (95% CI: 96.1–100)	*P*_A vs B_=0.56 *P*_A vs C_=0.56 *P*_B vs C_>0.99	94% (109/116) (95% CI: 88.1–97.1)	100% (120/120) (95% CI: 96.9–100)	99.2% (117/118) (95% CI: 95.4–100)	*P*_A vs B_=0.02 *P*_A vs C_ =0.07 *P*_B vs C_ =0.50
Serology at 12 weeks	98.7% (78/79) (95% CI: 93.2–99.9)	100% (80/80) (95% CI: 95.4–100)	100% (78/78) (95% CI: 95.3–100)	*P*_A vs B_=0.87 *P*_A vs C_>0.99 *P*_B vs C_>0.99	94.6% (106/112) (95% CI: 88.8–97.5)	100% (116/116) (95% CI: 96.8–100)	100% (114/114) (95% CI: 96.7–100)	*P*_A vs B_=0.04 *P*_A vs C_ =0.04 *P*_B vs C_ >0.99
Serology at 10 weeks	100% (59/59) (95% CI: 93.9–100)	100% (59/59) (95% CI: 93.9–100)	100% (57/57) (95% CI: 96.7–100)	*P*_A vs B_>0.99 *P*_A vs C_>0.99 *P*_B vs C_>0.99	95.1% (97/102) (95% CI: 89–97.9)	100% (105/105) (95% CI: 96.5–100)	100% (103/103) (95% CI: 96.4–100)	*P*_A vs B_=0.08 *P*_A vs C_ =0.08 *P*_B vs C_ >0.99
Serology at 8 weeks	100% (32/32) (95% CI: 89.3–100)	100% (31/31) (95% CI: 89–100)	100% (29/29) (95% CI: 88.3–100)	*P*_A vs B_>0.99 *P*_A vs C_>0.99 *P*_B vs C_>0.99	98.8% (85/86) (95% CI: 93.7–99.9)	100% (88/88) (95% CI: 95.8–100)	100% (86/86) (95% CI: 95.7–100)	*P*_A vs B_=0.87 *P*_A vs C_ >0.99 *P*_B vs C_ >0.99
Serology at 7 weeks	100% (22/22) (95% CI: 85.1–100)	100% (21/21) (95% CI: 84.5–100)	100% (19/19) (95% CI: 83.2–100)	*P*_A vs B_>0.99 *P*_A vs C_>0.99 *P*_B vs C_>0.99	98.7% (78/79) (95% CI: 93.2–99.9)	100% (80/80) (95% CI: 95.4–100)	100% (78/78) (95% CI: 95.3–100)	*P*_A vs B_ =0.87 *P*_A vs C_ >0.99 *P*_B vs C_ >0.99

^*a*^ High-avidity threshold (LIAISON), ≥0.350.

^*b*^ Sensitivity calculated as low and intermediate avidity/total percentage (*n/N*).

When considering the combined periconceptional and first-trimester period (Table 4), IgG avidity sensitivity would have been 100% with LIAISON, 94%–98.7% with Alinity i, and 99.2%–100% with VIDAS. LIAISON and VIDAS were significantly more sensitive than Alinity i when sampling occurred at 12 weeks of gestation (*P* = 0.04).

### Positive predictive value (PPV) of low or intermediate avidity for recent primary infection (less than 4 months)

The PPV of low IgG avidity for recent MPI was very high, reaching 100% (95/95) with VIDAS. VIDAS showed the highest PPV (Table 5), followed by LIAISON and then Alinity i (Table 5). In contrast, the PPV of intermediate avidity for recent MPI was low with all assays, with no significant difference between tests.

**TABLE 5:** Positive predictive value (PPV) at low or intermediate IgG avidity

Time between sample collection and maternal CMV primary infection	Alinity i CMV IgG avidity^*a*^ (A)	Alinity i CMV IgG avidity^*b*^ (B)	LIAISON CMV IgG Avidity II^*c*^ (C)	VIDAS CMV IgG Avidity II (D)	*P*-value
PPV at low IgG avidity					
≤90 days (3 months)	82.8% (101/122) (95% CI: 75.1–88.5)	94.4% (67/71) (95% CI: 86.4–97.8)	93.5% (86/92) (95% CI: 86.5–97)	100% (95/95) (95% CI: 96.1–100)	*P*_A vs C_ *=* 0.03 *P*_A vs D_ *<* 0.001 *P*_B vs C_ *=* 0.99 *P*_B vs D_ *=* 0.06 *P*_C vs D_ *=* 0.03
≤120 days (4 months)	86.1% (105/122) (95% CI: 78.8–91.1)	94.4% (67/71) (95% CI: 86.4–97.8)	95.7% (88/92) (95% CI: 89.4–98.3)	100% (95/95) (95% CI: 96.1–100)	*P*_A vs C_ *=* 0.04 *P*_A vs D_ *<* 0.001 *P*_B vs C_ *=* 0.73 *P*_B vs D_ *=* 0.09 *P*_C vs D_ *=* 0.06
PPV at intermediate IgG avidity					
≤90 days (3 months)	36.4% (4/11) (95% CI: 15.2–64.6)	61.3% (38/62) (95% CI: 48.9–72.4)	48.4% (30/62) (95% CI: 36.4–60.6)	40.4% (19/47) (95% CI: 27.6–54.7)	*P*_A vs C_ *=* 0.82 *P*_A vs D_ *>* 0.99 *P*_B vs C_ *=* 0.37 *P*_B vs D_ *=* 0.10 *P*_C vs D_ *=* 0.82
≤120 days (4 months)	45.5% (5/11) (95% CI: 21.3–72)	69.4% (43/62) (95% CI: 57–79.4)	58.1% (36/62) (95% CI: 45.7–69.5)	55.3% (26/47) (95% CI: 41.3–68.6)	*P*_A vs C_ *=* 0.89 *P*_A vs D_ *=* 0.93 *P*_B vs C_ *=* 0.46 *P*_B vs D_ *=* 0.41 *P*_C vs D_ *=* 0.93

^*a*^ Manufacturer’s threshold.

^*b*^ Low-avidity threshold, <30%; intermediate-IgG avidity threshold, ≥30% and <60%.

^*c*^ High-avidity threshold (LIAISON), ≥0.350.

Using a lower threshold to define low avidity with Alinity i (<30% instead of the manufacturer-recommended <50%) improved the PPV for recent MPI from 86.1% (105/122) to 94.4% (67/71) (Table 5).

### Benefit of confirmatory testing in cases of intermediate avidity

Because IgG avidity maturation may be differently appreciated between assays in semi-recent infections (≥4 months), we evaluated the benefit of retesting intermediate-avidity results with a second assay to confirm or exclude a recent MPI (≤4 months) (Fig. S1).

Among sera with intermediate-Alinity i avidity values (≥0.30 to <0.60), 18% (11/61) showed high avidity with VIDAS, allowing exclusion of recent MPI. Among sera with intermediate-VIDAS avidity values (≥0.50 to <0.65), 12.9% (4/31) showed high avidity with LIAISON.

Among sera with intermediate-LIAISON avidity values ranging from ≥0.150 to <0.250, 11.6% (5/43) showed high avidity with VIDAS, excluding recent MPI. For LIAISON intermediate-avidity values between ≥0.250 and <0.350, 57.9% (11/19) showed high avidity with VIDAS. Alinity i was not recommended as a second-line confirmatory assay as high-avidity results may be observed in patients with an MPI of <90 days. For intermediate-avidity values between ≥0.40 and <0.50 obtained with the VIDAS assay, a second-line confirmatory test is not recommended as it failed to effectively exclude recent MPI.

### Proposed decision-making algorithm in cases of positive or equivocal IgM detected during CMV serology screening in the first trimester of pregnancy

When IgM are positive or equivocal and IgG are positive, an IgG avidity assay is mandatory to date the infection. Based on the literature, high IgG avidity is expected in 70% to 80% of cases (6, 23, 24), excluding a MPI during the periconceptional and first-trimester periods with high sensitivity. Conversely, in 20%–30% of cases, avidity is low or intermediate, confirming or suggesting a suspicion of recent MPI. Based on the results of the present study, low IgG avidity demonstrates a high positive predictive value (PPV) for recent MPI, and intermediate avidity yields a poor PPV. Consequently, we propose confirming intermediate results using a second assay, as illustrated by “First assay” and “Second assay.” The choice of the confirmatory assay depends on the initial assay used and the intermediate index value obtained (see above and Fig. S1).

## DISCUSSION

The performance of CMV screening in the first trimester of pregnancy primarily depends on the sensitivity and specificity of the serological assays used. High sensitivity of both IgM and IgG avidity assays is essential to avoid missing detection of MPI occurring during the first trimester or the periconceptional period. At the same time, adequate specificity of IgG avidity assays for recent infection is crucial to accurately identify women who may benefit from valacyclovir prophylaxis. Indeed, the proportion of IgM positivity among pregnant women with a recent CMV primary infection (≤ 4 months) was significantly higher than in those with a later-stage infection (>4 months). Nevertheless, a substantial number of pregnant women maintained persistent IgM positivity for more than 4 months post-primary infection (Table S1). In France and Europe, four major manufacturers dominate the CMV serology market (Abbott, Roche, DiaSorin, and BioMérieux). In this study, we evaluated the performance of two IgM assays and three IgG avidity assays from three of these manufacturers, using a large panel of sera collected from pregnant women with a precisely dated MPI, sampled within 9 months of infection onset (median 62 days, range 9–252).

IgM sensitivity for detecting an MPI within the previous 3 to 4 months was high (93 to 100%) with both the Alinity i CMV IgM and the LIAISON CMV IgM II assays. Few studies have assessed IgM sensitivity using assays adapted to high-throughput automated systems and according to the time elapsed since MPI. Two of these studies showed that, depending on the assay used, IgM detection rates during the second and third months after MPI ranged from 86% to 97% and from 51% to 90%, respectively (25, 26). It should be noted in these studies that the Elecsys IgM CMV assay (Roche), which is one of the techniques used in automated high-throughput systems, was among the assays showing the lowest sensitivity (25, 26). One other study (27) reported a sensitivity of 90% for the LIAISON CMV IgM II assay in sera collected during the third month after MPI, which is lower than the 100% sensitivity observed in our study. This discrepancy is likely explained by differences in the interpretation of equivocal IgM results that were considered negative in this study (27), whereas they were considered positive in our analysis. This finding underscores the importance of performing IgG avidity testing not only in clearly IgM-positive sera but also in those with equivocal IgM results.

Projection of our data to first trimester and periconceptional screening scenarios showed excellent performance of the LIAISON IgM assay (>99%) irrespective of the sampling time. With the Alinity i assay, the sensitivity for detecting first-trimester MPI remained high (>96%) regardless of the sampling time but decreased to 92.9% for periconceptional MPI when sampling was performed late (14 weeks). These findings highlight the importance of performing CMV screening as early as possible during pregnancy to optimize IgM-based detection and ideally as soon as a pregnancy is known. IgG avidity sensitivity for detecting an MPI within the previous 3 or 4 months was very high (> 98%) with the LIAISON and VIDAS assays, whereas it was significantly lower with the Alinity i assay. Consistent with the literature, most recent-generation IgG avidity assays demonstrate high sensitivity (94%–100%) for excluding recent MPI (21, 26–30), although one study also reported a lower sensitivity of the Alinity i avidity assay (19). One of this study (27) reported a sensitivity of 94% for the LIAISON CMV IgG Avidity II assay in sera collected during the third month after MPI, which is lower than the 100% observed in our study. This difference is explained by the avidity threshold used in this study (27), which applied the manufacturer-recommended high-avidity threshold of 0.250, whereas we used a higher threshold of 0.350. Indeed, results from these two studies (12, 13) suggested that the threshold value recommended by the manufacturer for high avidity (>0.250) with the LIAISON CMV IgG Avidity II is too low. For example, in one of these studies (5), 10 women with primary infection in their first trimester and LIAISON avidity between 0.250 and 0.350 transmitted the virus to their fetus. In the present study, applying the threshold of 0.250 to our data, LIAISON would decrease the sensitivity for detecting MPI within 3 months to 97.4%.

The projected performance for first trimester and periconceptional screening showed excellent sensitivity with both the LIAISON and VIDAS assays (>99%), regardless of the sampling time. With the Alinity i assay, the sensitivity remained high (>97%) for first-trimester MPI but dropped below 95% for periconceptional MPI when the sampling was performed after 10 weeks.

The positive predictive value of low IgG avidity for very recent MPI (<3 months) was excellent with VIDAS and LIAISON and lowest with Alinity i (100%, 93.5%, and 82.8%, respectively). However, restricting Alinity i low avidity to values < 30% improved the PPV to 94.4%. In contrast, the PPV of intermediate avidity for recent MPI was low across all assays (36.4%–48.4%; Table 5), indicating that approximately half of women with positive IgM and intermediate avidity do not have a recent MPI and therefore carry a substantially lower risk of vertical transmission.

**Fig 1:**
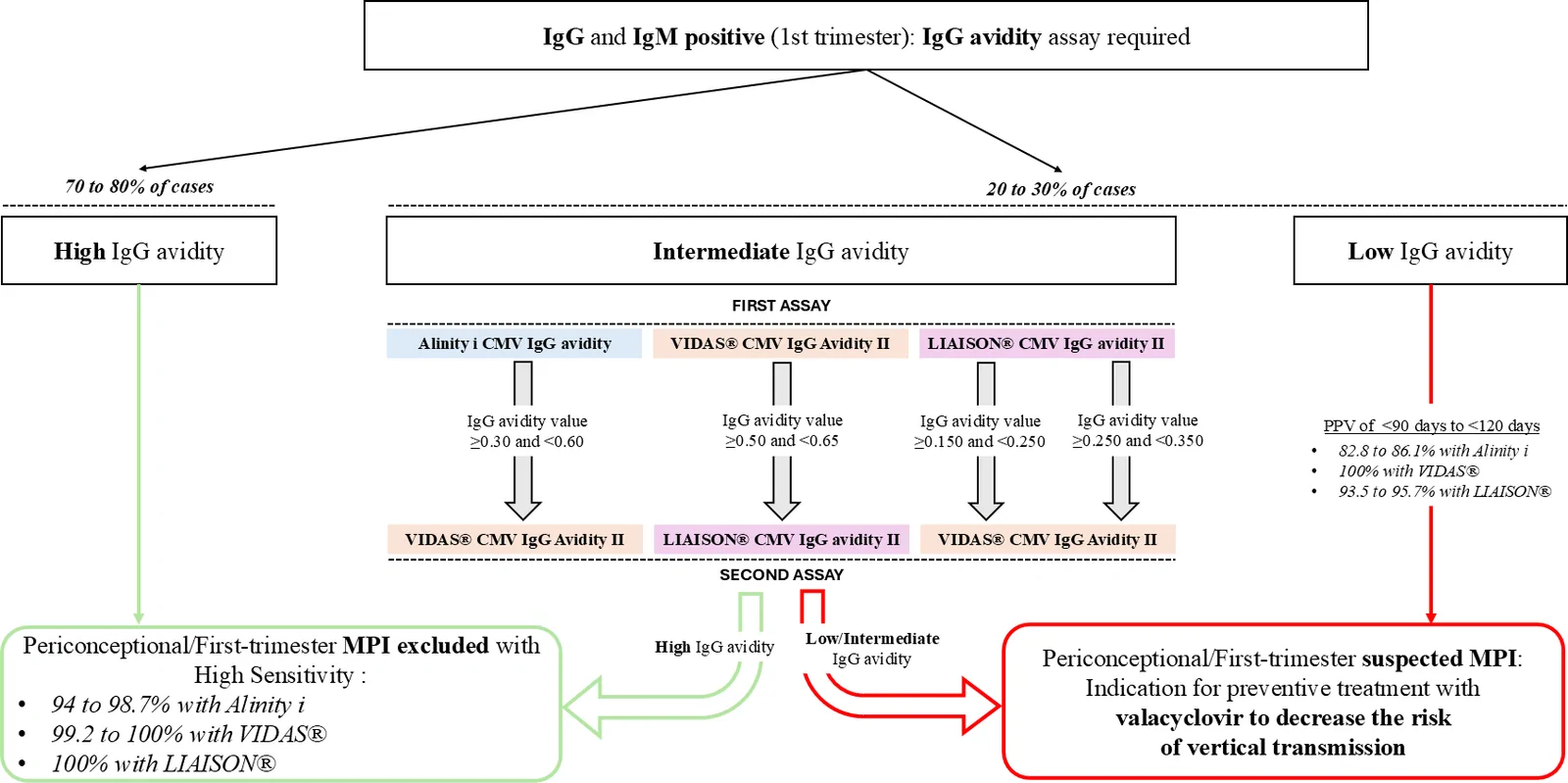
Proposed decision-making algorithm in cases of positive or equivocal IgM detected during CMV serology screening in the first trimester of pregnancy.

Because IgG maturation over time may be interpreted differently by distinct avidity assays, we assessed the value of confirmatory testing with a second assay in cases of intermediate avidity. We found that retesting sera with intermediate avidity using an alternative assay (VIDAS or LIAISON) could meaningfully improve the classification of recent versus non-recent MPI, particularly for intermediate results obtained with VIDAS (≥0.50 to <0.65), LIAISON (0.150–0.350), or Alinity i (0.30–0.60) (Fig. 1).

### Strengths and limitations

The main strength of this study lies in the large number of sera obtained from pregnant women with a precisely dated and well-characterized primary CMV infection. A limitation of the study is its single-center design. Due to the limited amount of serum available, only 92% (162/176) of sera could be tested using all three avidity assays, potentially introducing a bias in the analysis. Another limitation is that only two IgM assays and three IgG avidity assays were evaluated. However, these assays represent the main tools used in clinical practice: in France, approximately 50% of laboratories use either the LIAISON or Alinity i IgM assays, and virtually all laboratories rely on VIDAS, LIAISON, or Alinity i for CMV IgG avidity testing.

### Conclusion

Both Alinity i and LIAISON IgM assays are sufficiently sensitive to be used as screening tools for primary CMV infection during the first trimester of pregnancy and the periconceptional period. The three avidity assays are reliably able to exclude a first-trimester MPI regardless of the sampling time. However, when sampling is performed after 10 weeks, VIDAS and LIAISON avidity assays are significantly more sensitive than Alinity i for detecting periconceptional MPI. Future studies should focus on evaluating the performance of other IgM and avidity tests used on high-throughput automated systems to determine their suitability for routine screening for CMV during the first trimester of pregnancy.

## Data Availability

The data presented in this study are available from the corresponding author upon reasonable request.

## SUPPLEMENTAL MATERIAL

Figure S1 jcm.00545-26-s0001.pdf Detailed figure showing the results of confirmatory testing for cases with intermediate avidity at first-line screening. First-line IgG avidity assays are shown in the orange rectangle. Second-line IgG avidity assays performed on samples with intermediate avidity in the first-line assays are shown in the blue rectangle. The McNemar test was used for pairwise comparisons.

Table S1 jcm.00545-26-s0002.pdf The proportion of IgM anti-CMV positivity in pregnant women with a recent maternal CMV primary infection (≤4 months) and with an old CMV primary infection (>4 months). Fisher's exact test was used for pairwise comparisons. Equivocal results were classified as positive.

ASM does not own the copyrights to Supplemental Material that may be linked to, or accessed through, an article. The authors have granted ASM a non-exclusive, world-wide license to publish the Supplemental Material files. Please contact the corresponding author directly for reuse.
