# Supplementary material for: Comparative performance of CMV IgM and IgG avidity assays for diagnosing a recent primary infection in the first trimester of pregnancy
Source: J Clin Microbiol. 2026 Aug 10;64(9):e00545-26. doi: 10.1128/jcm.00545-26 (PMC13556237; doi:10.1128/jcm.00545-26)
Supplement: Supplementary file 1 [file jcm.00545-26-s0001.pdf]

Supplementary Figure 1. Detailed figure showing the results of confirmatory testing for cases with intermediate avidity at first-line screening
First-line IgG avidity assays are shown in the orange rectangle.
Second-line IgG avidity assays performed on samples with intermediate avidity in the first-line assays are shown in the blue rectangle.
The McNemar test was used for pairwise comparisons.

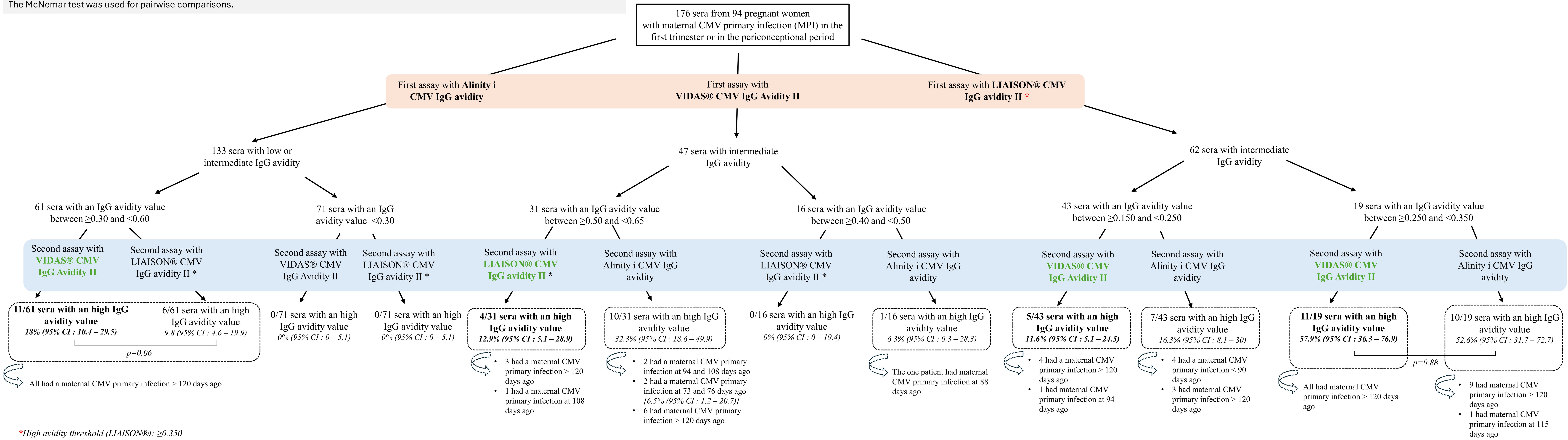

*High avidity threshold (LIAISON®): ≥0.350
