# Supplementary material for: Comparative performance of CMV IgM and IgG avidity assays for diagnosing a recent primary infection in the first trimester of pregnancy
Source: J Clin Microbiol. 2026 Aug 10;64(9):e00545-26. doi: 10.1128/jcm.00545-26 (PMC13556237; doi:10.1128/jcm.00545-26)
Supplement: Supplementary file 2 [file jcm.00545-26-s0002.pdf]

The proportion of IgM anti-CMV positivity in pregnant women

	with a **recent** maternal CMV primary infection (**≤ 4 months**)	with an **old** maternal CMV primary infection (**> 4 months**)	
**Alinity i CMV IgM Sensitivity % (n/N)[§]**	**92.2 %** (94/102) (95% CI : 85.3 – 96.0)	**65.9 %** (29/44) (95% CI : 51.1 – 78.1)	P=0.0003
**LIAISON® CMV IgM II Sensitivity % (n/N)[§]**	**99.2 %** (122/123) (95% CI : 95.5 - 100)	**92.0 %** (46/50) (95% CI : 81.2 – 96.8)	P=0.0250

[§]Sensitivity calculated as equivocal and positive CMV IgM /total %, (n/N).

Supplementary Table 1. The proportion of IgM anti-CMV positivity in pregnant women with a recent maternal CMV primary infection (≤ 4 months) and with an old CMV primary infection (> 4 months).
The Fisher's Exact test was used for pairwise comparisons. Equivocal results were classed as positive.
